# REDOX Balance in Oligodendrocytes Is Important for Zebrafish Visual System Regeneration

**DOI:** 10.3390/antiox12122026

**Published:** 2023-11-22

**Authors:** Cristina Pérez-Montes, Jhoana Paola Jiménez-Cubides, Almudena Velasco, Rosario Arévalo, Adrián Santos-Ledo, Marina García-Macia

**Affiliations:** 1 Instituto de Neurociencias de Castilla y León (INCyL), 37007 Salamanca, Spain; cristina_perez_montes@usal.es (C.P.-M.); jhoanajicu@usal.es (J.P.J.-C.); malmu@usal.es (A.V.); mraa@usal.es (R.A.); 2 Department of Human Anatomy and Histology, Universidad de Salamanca, 37007 Salamanca, Spain; 3 Instituto de Investigación Biomédica de Salamanca (IBSAL), 37007 Salamanca, Spain; 4 Department of Cell Biology and Pathology, Universidad de Salamanca, 37007 Salamanca, Spain; 5 Institute of Functional Biology and Genomics (IBFG), Universidad de Salamanca/CSIC, 37007 Salamanca, Spain; 6 Department of Biochemistry and Molecular Biology, Universidad de Salamanca, 37007 Salamanca, Spain; 7 Centre for Biomedical Investigations Network on Frailty and Ageing (CIBERFES), 28029 Madrid, Spain

**Keywords:** melatonin, regeneration, ROS, zebrafish, visual system

## Abstract

Zebrafish (*Danio rerio*) present continuous growth and regenerate many parts of their body after an injury. Fish oligodendrocytes, microglia and astrocytes support the formation of new connections producing effective regeneration of the central nervous system after a lesion. To understand the role of oligodendrocytes and the signals that mediate regeneration, we use the well-established optic nerve (ON) crush model. We also used *sox10* fluorescent transgenic lines to label fully differentiated oligodendrocytes. To quench the effect of reactive oxygen species (ROS), we used the endogenous antioxidant melatonin. Using these tools, we measured ROS production by flow cytometry and explored the regeneration of the optic tectum (OT), the response of oligodendrocytes and their mitochondria by confocal microscopy and Western blot. ROS are produced by oligodendrocytes 3 h after injury and JNK activity is triggered. Concomitantly, there is a decrease in the number of fully differentiated oligodendrocytes in the OT and in their mitochondrial population. By 24 h, oligodendrocytes partially recover. Exposure to melatonin blocks the changes observed in these oligodendrocytes at 3 h and increases their number and their mitochondrial populations after 24 h. Melatonin also blocks JNK upregulation and induces aberrant neuronal differentiation in the OT. In conclusion, a proper balance of ROS is necessary during visual system regeneration and exposure to melatonin has a detrimental impact.

## 1. Introduction

Regenerative medicine aims to regain the lost functionality of an organ due to trauma, injury or disease using organ specific mechanisms. Unfortunately, in most cases, these mechanisms are not properly elucidated [1]. In humans, some tissues, such as blood and epithelia, regenerate efficiently; while others, such as the heart and nervous system, do not. Many groups have devoted themselves to repairing injuries in the nervous system using prosthetics [2], stem cells [3] and gene therapy [4]; their success has been very limited and, so far, there is no efficient regenerative treatment. Unlike mammals, zebrafish (*Danio rerio*) present a robust regenerative capacity, including of the nervous system [5,6]. Furthermore, zebrafish are currently used to model a wide range of human diseases where regenerative therapies could be applied, from heart diseases to Alzheimer’s [7,8]. Understanding the regenerative mechanisms in zebrafish would facilitate strategies for regeneration in humans.

The zebrafish visual system resembles that of a human. It is formed by the retina, the optic nerve (ON), formed by the axons of the ganglion cells, and the superior visual center, that in fish is the optic tectum (OT). Gross injuries in the visual system, such as strong light, cryoinjury of the retina and ON crush, cause the degeneration of the ON. Then, the retina loses the connection with the OT, leading to partial and temporal blindness [9]. In zebrafish, many retinal ganglion cells survive the injury and create new axons that are projected contralaterally towards the OT and vision is restored [10]. Glial cells are very important during regeneration and one key difference between mammals and zebrafish is that, in the former, glia do not form a scar but create a regenerative-permissive environment [1,11,12]. Furthermore, in zebrafish during the degeneration and posterior regeneration of oligodendrocytes, the myelinating cells of the central nervous system (CNS) play a crucial role [5]. After injury, many oligodendrocytes die, and new ones arise from oligodendrocyte progenitor cells. These newly formed oligodendrocytes create myelin as the axons regenerate [5]. Mature oligodendrocytes can also survive degeneration and contribute to myelination [13]. However, the role of these latter oligodendrocytes is more controversial, and they have been described to produce aberrant myelination [14]. The role of the oligodendrocytes during regeneration has been well explored in zebrafish larvae [14] and in the adult spinal cord [12] but their exact role in the visual system remains more obscure.

Another open question relates to the cues that are needed for proper regeneration. Certainly, communication between neurons, glia and other cells such as macrophages is key [15]. Several extrinsic and intrinsic signals, such as synaptic sensing, vesicle release and neurotransmitters, have been shown to contribute during regeneration [5,16]. Reactive oxygen species (ROS) have also emerged as relatively new players in this context [17]. ROS are highly reactive chemicals produced from O_2_ as normal biproducts of the mitochondrial metabolism and membrane NADPH oxidase. This includes the superoxide ion (O_2_^−^) that is the precursor of many others such as hydrogen peroxide (H_2_O_2_). ROS are short-lived but, thanks to their small size, some of them can freely diffuse among cells, playing important roles as signaling molecules [18]. ROS are involved in cell communication, proliferation, cell death and can promote, among other processes, oligodendrogenesis [19]. In zebrafish, ROS are produced after tail injury and promote its regeneration [20] and the presence of antioxidants can be detrimental to an efficient regeneration [21]. CNS is no exception to the production of ROS after injury, but its impact is less well understood [22]. For example, in some contexts, such as demyelinating neurological diseases, excessive ROS are detrimental [23]. Free radicals are a very interesting puzzle since they can exert both beneficial and detrimental effects.

We explore the role of ROS during zebrafish visual system regeneration, using the ON crush model, specifically in fully differentiated oligodendrocytes. To explore the beneficial or detrimental role of oxidative stress, we treated the fish with melatonin, which is a well-known endogenous antioxidant [24,25]. This molecule has been successfully used to reverse cognitive and endocrine deficits produced by alterations of the zebrafish visual system [26].

## 2. Methods

### 2.1. Animals

A total of 112 animals were used, half males and half females: 4 animals for each group and treatment in every experiment. All fish used in this work were 12 months old (adults). Fish were kept under a 14 h light–10 h dark cycle and fed twice a day. The water temperature was maintained at 27.8 ± 0.5 °C; pH 7.0 ± 0.2. Animals were fed before surgery and before changing the melatonin solution. We employed two transgenic lines to track mature oligodendrocytes: *Tg(sox10:EGFP)* [27] and *Tg(sox10:tagRFP)* [28]. These lines carry either EGFP or tagRFP under the control of *sox10*. This transcription factor is involved in the differentiation of oligodendrocytes, becoming active when they turn into myelinating cells [29,30,31]. For the cytometry flux experiments we used the EGFP line because it does not cross with the probes’ fluorescence. For the immunohistochemistry experiments we used the tagRFP line because it does not require amplification by immunohistochemistry. All lines were kindly donated by Bruce Appel.

All protocols were performed according to the European Union Directive 86/609/EEC and Recommendation 2007/526/EC, regarding the protection of animals used for experimental and other scientific purposes, enforced in Spanish legislation under the law 6/2013. All protocols were approved by the Bioethics Committee of the University of Salamanca.

### 2.2. Optic Nerve Crush

ON crush was performed as previously described [32]. Briefly, fish were anesthetized in tricaine methane sulfonate (MS-222) (Sigma-Aldrich, St. Louis, MO, USA) and positioned under a stereomicroscope on an ice-cold surface. A fine forcep was used to partially pull the temporal half of the right eye out of its orbit. Then, the lateral rectus eye muscle was cut with a fine scissor and the eye was pulled out to further expose the ON. With another fine forceps, we crushed the ON, avoiding the ophthalmic artery running along the ON. The fish successfully operated on were return to a fish tank, and allowed to survive for 3, 24 and 72 h after the lesion. The same protocol without crushing was applied to control animals.

### 2.3. Melatonin Treatment

Exposure to melatonin was performed by immersion, as previously described [26]. Melatonin (98%, Sigma-Aldrich, Saint Louis, MI, USA) was dissolved in ethanol, creating a stock solution of 2.32 mg/mL. Stock solution was wrapped in foil and stored at −20 °C in the dark until use. Melatonin solution at 0.232 mg/L was prepared fresh every time in a 0.5 L tank with standard system water. Control and injured fish with no melatonin were exposed to 0.01% ethanol as the vehicle. One animal was allocated per tank. Thus, the experimental groups were set as control 3, 24 and 72 h (+0.01% ethanol for that period); control melatonin 3, 24 and 72 h (+0.232 mg/L melatonin for that period), regeneration 3, 24 and 72 h (+0.01% ethanol for that period) and regeneration melatonin 3, 24 and 72 h (+0.232 mg/L melatonin for that period).

Zebrafish are diurnal animals linked to circadian rhythms that are modulated by melatonin, so we were careful with the timing of our experiments [25]. Lesion was always performed at 9 am when melatonin production is lowest, followed by exposure to vehicle or melatonin. A fresh solution containing either vehicle or melatonin was prepared every time. In the case of the 3 h group, sacrificed was performed at 12 am. In the case of the 24 h group, the solution was changed once at 9 pm and the fish were sacrificed the following day at 9 am. In the case of the 72 h group, the solution was changed twice every day (9 am and 9 pm) to ensure the stability of the melatonin [26,33], and the fish were sacrificed three days later at 9 am.

### 2.4. Brain Dissection and Tissue Processing

At 3, 24 and 72 h, fish were endpoint anesthetized in tricaine methane sulfonate before sacrifice, according to Spanish and European laws (2010/63/EU; RD 53/2013; Ley 32/2007; and Orden ECC/566/2015). The head was removed, and the entire brain, including the eyes and ON, was carefully dissected manually with forceps in cold PBS under a microscope.

To prepare the brain for the histochemistry and the immunostaining techniques, the tissue was fixed in paraformaldehyde 4% overnight at 4 °C. The sample was cryoprotected in 10% sucrose in PBS and embedded in a solution containing 1.5% agar and 10% sucrose. Finally, 12 mm horizontal sections, to observe ON and OT simultaneously, were obtained in a cryostat. Sections were stored at −20 °C until use.

For flow cytometry we used a modified protocol [34]. Briefly, both ON, the ipsilateral OT (IOT) and the contralateral OT (COT), were sectioned with surgical scissors and located separately in solution A (EBBS, 2% BSA, 1.3% DNase) in ice. After collecting the tissue from all animals, tissue was transferred to solution B (EBBS medium, 2% BSA, 1.3 DNase, 1% Trypsine). Tissue was incubated for 5 min at 37 °C, then disaggregated with a siliconized pipette and then incubated for 10 more minutes. Samples were centrifuged for 5 min at 3000× *g* at 4 °C, supernatant was removed and 1 mL of solution A was added. Sample was resuspended with a blue tip and centrifuged again. Finally, supernatant was removed and 1 mL of Hank’s solution plus 200 μL of KRPG buffer (145 mM NaCl, 5.7 mM, 566 Na_2_HPO_4_, 4.86 mM KCl, 0.54 mM CaCl_2_, 1.22 mM MgSO_4_, 5.5 mM glucose, pH 7.35) were added and created a cell suspension.

### 2.5. Probes and Flow Cytometry

Mitochondrial ROS were determined with the fluorescent probe MitoSox^®^ (Life Technologies, Carlsbad, CA, USA) [34]. Cell suspensions were incubated with 2 μM of MitoSox^®^ for 30 min at 37 °C in a 5% CO_2_ atmosphere in KRPG buffer. MitoSox^®^ fluorescence intensity was assessed by flow cytometry (FACScalibur flow cytometer, BD Biosciences, Franklin Lakes, NJ, USA) and expressed in arbitrary units.

The mitochondrial membrane potential (Δψm) was assessed with MitoProbe DiIC1^®^ (Life Technologies) (50 nM) by flow cytometry (FACScalibur flow cytometer, BD Biosciences) and expressed in arbitrary units [34]. For this purpose, cell suspensions were incubated with a probe 30 min at 37 °C in PBS. Values of Δψm were obtained after subtraction of the potential value determined in the presence of carbonyl cyanide-4-(trifluoromethoxy) phenylhydrazone (CCCP) (10 μM, 15 min) for each sample.

### 2.6. Histochemistry

Acetylcholine esterase (AChE) histochemistry was performed as previously described [35]. Briefly, sections were rehydrated in 0.1 M maleate buffer pH 6.0, and then incubated at room temperature for 30 min in a medium containing 65 mM maleate buffer pH 6.0, 0.5 mM sodium citrate, 0.47 mM cupric sulphate, 0.05 mM potassium ferricyanide, 0.37 mM acetylthiocholine iodide and 0.06 mM ethopropazine as an inhibitor of non-specific esterases. AChE activity was enhanced using 0.0125% DAB and 0.0033% H_2_O_2_ in 0.2 M Tris-HCl buffer, pH 7.6. The reaction was monitored under the microscope and stopped by washing the sections in the same Tris-HCl buffer (around 45 min). Finally, sections were dehydrated in an ethanol series, cleared with xylene and cover slipped with Entellan.

### 2.7. Immunostaining

Sections were washed several times in PBS and then pre-incubated for 90 min in 5% normal donkey (DK) serum in PBS with 0.2% Triton X-100 (PBSTx) at room temperature (RT). After that, primary antibodies (Table 1) were incubated overnight in 5% normal DK serum with 0.2% PBSTx at 4 °C. Sections were washed in PBS and then incubated for 90 min at RT in darkness with a 1:400 dilution of Alexa 488 and Alexa 647 fluorescent secondary antibodies (Table 2), in a buffer containing 5% normal DK serum in PBS. Next, sections were washed in PBS and then incubated for 10 min in 1:10,000 4′,6-diamidino-2-fenilindol (DAPI) for nucleus staining. Sections were washed thoroughly and mounted with Fluoromount-G^®^ Mounting Medium (Invitrogen, Waltham, MA, USA). The RFP fluorescence in the *sox10:tagRFP* transgenic line was robust enough and we never used an antibody to reinforce it.

### 2.8. Western Blot

OT cells were lysed in a commercial RIPA Lysis buffer (0.1% sodium dodecylsulfate, 1% NP-40, 150 mM NaCl, 1% sodium deoxycholate and 25 mM Tris-HCl, pH 7.6) (G-Biosciences). ON cells were lysed in a noncommercial RIPA buffer (1% sodium dodecylsulfate, 1% NP-40, 150 mM NaCl, 5% sodium deoxycholate and 25 mM Tris-HCl, pH 8). Lysis buffers were supplemented with a protease and phosphatase inhibitor mix (Sigma-Aldrich). Aliquots of cell lysates (10–20 μg of protein from ON or OT, respectively) were subjected to SDS/PAGE 10–12% (vol/vol) acrylamide gel (MiniProtean; Bio-Rad, Hercules, CA, USA) including PageRuler Prestained Protein Ladder (Thermo Fisher Scientific, Waltham, MA, USA). The resolved proteins were transferred electrophoretically to nitrocellulose membranes (0.2 μm, BioRad). Membranes were blocked with 5% (wt/vol) low-fat milk in TTBS (20 mM Tris, 150 mM NaCl, and 0.1% (vol/vol) Tween 20 (pH 7.5) for 1 h. After blocking, membranes were immunoblotted with primary antibodies, anti-p-JNK and JNK (1/1000, Cell Signalling, Danvers, MA, USA) and MBP (Myelin basic protein, 1/500, Abcam, Cambridge, United Kingdom) overnight at 4 °C. After incubation with horseradish peroxidase conjugated anti-rabbit IgG (1/5000, Cell Signalling), membranes were immediately incubated with the Supersignal West Femto (Thermo Fisher Scientific) before exposure using the Fusion FX transilluminator (Vilber GmbH, Eberhardzell, Germany).

### 2.9. Imaging and Quantification

AChE histochemistry was imaged in a light microscope (Leica Aristoplan; Leica, Wetzlar, Germany) with an Olympus OP- 70 digital camera (Olympus Corporation, Shinjuku City, Tokyo, Japan) coupled to an Olympus Provis AX70 photomicroscope using a 20× objective. All sections were imaged in the same session to avoid variations. To quantify the staining, FIJI 2.9.0 software and the threshold plugin were used. Images were turned into black and white, inversed and turned into 8 bits. Then threshold was established based on control animals to 110/200. Integrated density and area were measured for every section. Three non-consecutive sections were quantified from four different fish and the average is shown in the graphs.

ChAT and SDHB immunostainings were imaged in a Confocal microscope (Leica Stellaris inverted DMi8) using a 40× oil immersion objective for ChAT and 63× oil immersion objective for SDHB. For ChAT, 6 tiles with z-stack (xyz scan) were acquired and automatically assembled by the Leica LASX software. For SDHB, 4 tiles with z-stack were acquired and automatically assembled by the LASX software. The number of z-stacks was determined by observing the limits of each section (14 µm). Images were obtained under constant conditions to minimize image acquisition variation and stored as 1024 × 1024 pixels and 8-bit TIFF files.

Colocalization between SDHB and *sox10:tagRFP* oligodendrocytes was analyzed using FIJI, as previously described [36]. First, background from the *sox10:tagRFP* channel and SDHB channel was subtract with the “subtract background” tool (rolling ball radius 100 pixels). Then, *sox10:tagRFP* oligodendrocytes were selected manually with the “polygon selections” tool and added to ROI Manager. The SDHB threshold was established based on control animals and added to ROI Manager. To analyze the colocalization between the *sox10:tagRFP* and SDHB channels, each *sox10:tagRFP* oligodendrocyte selection was merged with the SDHB threshold using the command “AND” from ROI Manager. Then, merged selections were added to ROI Manager one by one. Finally, area, integrated density and mean gray value were measured for every merged selection added to ROI Manager using the command ‘’Measure’’ from the ROI Manager. Eight cells (four from tectal OT and four from the periventricular gray zone, PGZ) from three non-consecutive sections from four different animals in each group were used.

*Sox10:tagRFP* oligodendrocytes were quantified manually using the Cell Counter plugin from FIJI in the tectal OT and PGZ. Three non-consecutive sections from four different animals in each group were used.

In case of ChAT immunohistochemistry, positive neurons were quantified manually using the Cell Counter plugin from FIJI in the middle portion of the OT. Three non-consecutive sections from four different animals in each group were used. The average of these three sections is shown in the graphs.

The protein abundances of all Western blots per condition were measured by densitometry of the bands quantified with FIJI in the linear phase of the exposure without reaching saturation. At least three biologically independent replicates were always performed, although only one representative Western blot is usually shown in the main figures.

Figures were generated using Adobe Photoshop CS6.

### 2.10. Statistics

Graphs were generated using GraphPad Prism 5. Data are represented as mean ± standard error of mean (SEM). Student’s *t*-test was performed when comparing two groups (for example regeneration vs. control). ANOVA with Tukey´s Multiple Comparison Test was performed to search for differences among all groups (for example, the number of oligodendrocytes at any specific timepoint). The type of statistics and the significance are included in the figure legends.

## 3. Results

### 3.1. ROS Are Produced by sox10:EGFP Oligodendrocytes after Optic Nerve Crushing

**Figure 1:**
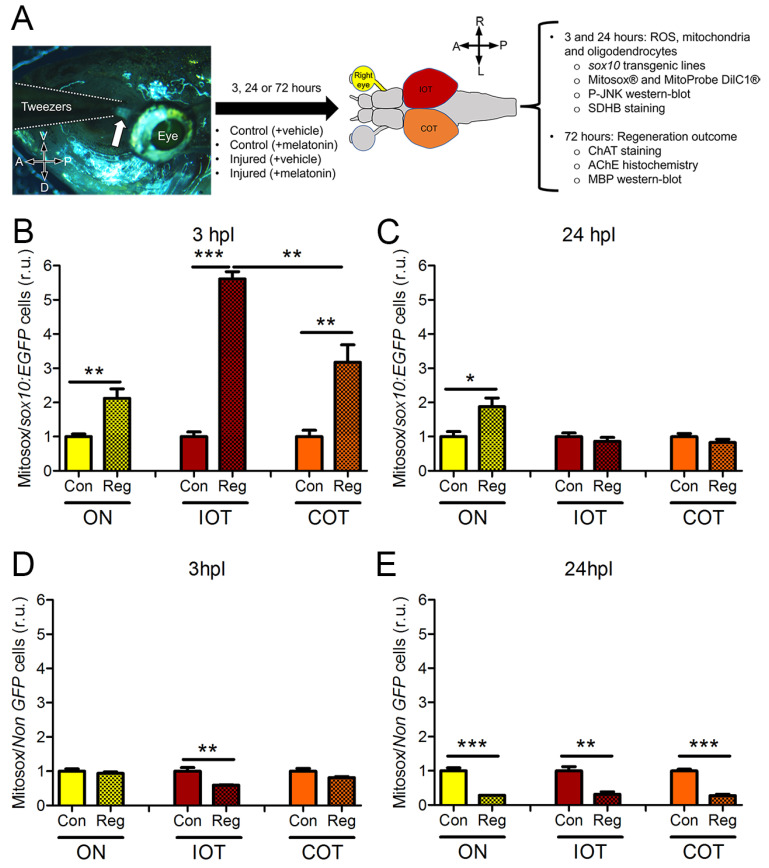
ROS measurement after ON crush. (**A**) Experimental design; the arrow points to the crushed ON from the ventral side. (**B**–**E**) Mitosox^®^ fluorescence quantified by flow cytometry in *sox10:EGFP* cells at 3 hpl (**B**) and 24 hpl (**C**) or *non-GFP* cells at 3 hpl (**D**) and 24 hpl (**E**). Values are relative to control mean. A: anterior; Con: control; COT: contralateral optic tectum; D: dorsal; hpl: hours post-lesion; IOT: ipsilateral optic tectum; L: left; ON: optic nerve; P: posterior; R: right; Reg: regeneration; r.u.: random units; V: ventral. Student’s *t*-test between Reg and Con for each timepoint and region * *p* < 0.05; ** *p* < 0.01; *** *p* < 0.001. n = 4 for each group.

The role of ROS during regeneration has been shown in several scenarios [20,22]. To understand whether they are also involved during zebrafish visual system regeneration, we used the ON crush model [32]. The right ON was always injured. After letting the fish survive for 3, 24 or 72 h, we extracted the whole brain together with the eyes and dissected three different parts of the visual system: both ON together, the ipsilateral optic tectum (IOT, right one) and the contralateral optic tectum (COT, left one). In zebrafish all fibers from ON cross the midline and reach the opposite side [37]. Thus, COT is the side that lost the projections. We disaggregated the cells and exposed them to Mitosox^®^, a probe that responds to O_2_^−^ (Figure 1A). Then, we run the cells through flow cytometry, sorting the *sox10:EGFP* cells (fully differentiated oligodendrocytes).

In the three visual areas investigated, sox10:EGFP oligodendrocytes show a greatly increased ROS production at 3 hpl (Figure 1B). The IOT presented the highest peak, with almost six times more ROS production than the control fish, followed by COT that tripled ROS (Figure 1B). These changes were not due to differences in the mitochondrial population state, as indicated by the lack of differences in the membrane potential when measured with MitoProbe DiIC1^®^ (Figure S1A). At 24 hpl, the effect on ROS production passed in the OT but it was maintained in the ON (Figure 1C). *Non-GFP* cells behaved differently (Figure 1D). *Non-GFP* cells at 3 hpl showed a reduction in ROS production but only in the IOT, the side that is not injured and, thus, is responding to the healthy ON (Figure 1D). At 24 hpl, *non-GFP* cells from the three areas showed a reduction in ROS. This analysis shows that there is an important but small window of ROS production in *sox10:EGFP* oligodendrocytes.

### 3.2. Melatonin Does Not Always Exert an Antioxidant Effect in sox10:EGFP Oligodendrocytes

**Figure 2:**
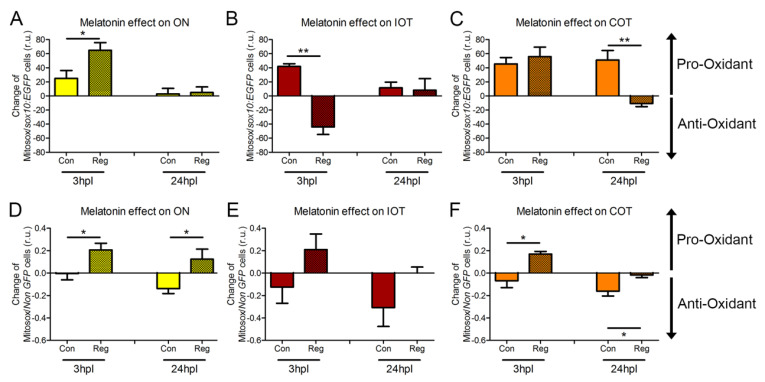
Melatonin impact on ROS production. (**A**–**F**) Mitosox^®^ fluorescence quantified by flow cytometry in the *sox10:EGFP* cells of ON (**A**), IOT (**B**) and COT (**C**) and in the *non-GFP* cells of ON (**D**), IOT (**E**) and COT (**F**) at 3 and 24 hpl. The value shown in the graph is the result of the ROS measurement in each animal treated with melatonin minus the average ROS value in animals treated with vehicle in each respective group. Con: control; COT: contralateral optic tectum; hpl: hours post-lesion; IOT: ipsilateral optic tectum; Mel: melatonin; ON: optic nerve; Reg: regeneration; r.u.: random units. For (**A**–**F**), Student’s t-test was performed between Reg and Con for each timepoint and region. * *p* < 0.05; ** *p* < 0.01. n = 4 for each group in all experiments.

To explore whether ROS played a positive or negative role during regeneration of the visual system, we exposed the zebrafish to the natural antioxidant melatonin. Since melatonin was dissolved in ethanol, control animals were also treated with this vehicle. Therefore, for every timepoint we have four experimental groups (see Figure 1A for more details). In the graphs, we show the change in ROS production after exposing the fish to melatonin for all groups. The value shown in the graph (Figure 2) is the result of the ROS measurement in each animal treated with melatonin minus the average ROS value in animals treated with vehicle in each respective group. Thus, positive values mean higher production of ROS after melatonin treatment (pro-oxidative) and negative ones mean a reduction (antioxidant).

Surprisingly, exposure to melatonin induced the production of mitochondrial ROS (pro-oxidant) in *sox10:EGFP* oligodendrocytes at 3 hpl in the ON of both control and injured fish, with more production in the latter. (Figure 2A). This effect was not observed at 24 h (Figure 2A). In the IOT, melatonin had an antioxidant effect in the regenerating fish but not in the control fish at 3 h, an effect that was absent at 24 h (Figure 2B). The opposite happened in the COT, the side that is primary responding to the injury: at 3 h, melatonin equally increased ROS production in the COT of the control and regenerating fish but we observed an antioxidant effect in the COT of the regenerating fish at 24 h (Figure 2C).

In all three areas at both timepoints, *non-GFP* cells had a reduced production of ROS after melatonin treatment in control animals (Figure 2D–F). In the regenerating fish, *non-GFP* cells either did not respond (IOT and COT at 24 hpl, Figure 2E,F) or the production was slightly increased (ON at 3 and 24 hpl, and IOT and COT at 3 hpl, Figure 2D–F).

### 3.3. Oligodendrocytes and Their Mitochondrial Population Are Altered during Regeneration

**Figure 3:**
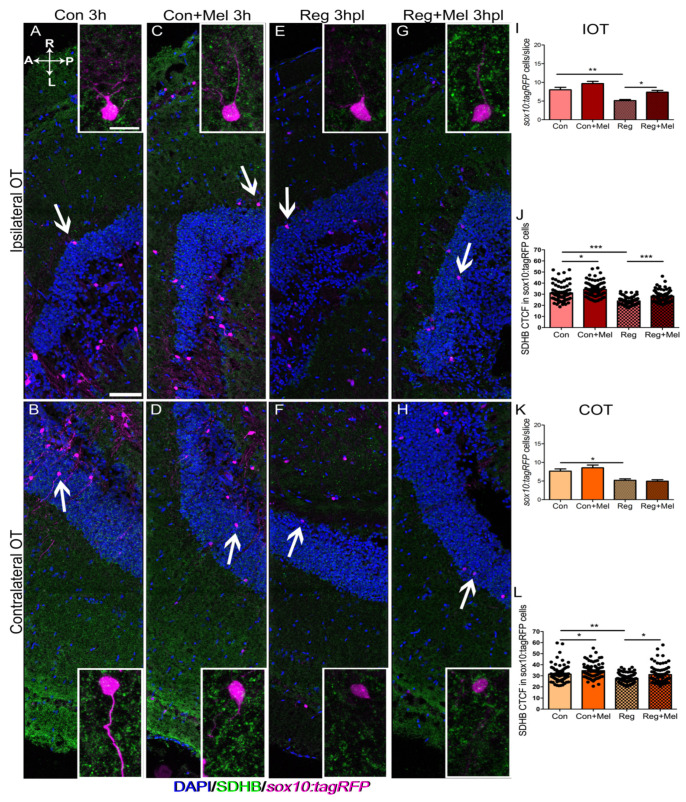
Analysis of oligodendrocyte and the mitochondrial distribution at 3 hpl. Immunostaining for SDHB (green) in a transgenic line carrying *sox10:tagRFP* reporter (magenta). Nuclei are counterstained with DAPI (blue). (**A**–**H**) Control IOT (**A**), control COT (**B**), control exposed to melatonin IOT (**C**), control exposed to melatonin COT (**D**), regenerating fish IOT (**E**), regenerating fish COT (**F**), regenerating fish exposed to melatonin IOT (**G**), regenerating fish exposed to melatonin COT (**H**). (**I**–**L**) Quantification of the number of oligodendrocytes per section in the IOT (**I**) and the COT (**K**); CTCF value of SHDB in oligodendrocytes in the IOT (**J**) and the COT (**L**). A: anterior; Con: control; COT: contralateral optic tectum; h: hours; hpl: hours post-lesion; IOT: ipsilateral optic tectum; L: left; Mel: melatonin; P: posterior; R: right; Reg: regeneration; r.u.: random units. ANOVA with Tukey’s Multiple Comparison Test * *p* < 0.05; ** *p* < 0.01; *** *p* < 0.001. Scale bar: 100 mm; 10 mm in inset. n = 4 for each group.

**Figure 4:**
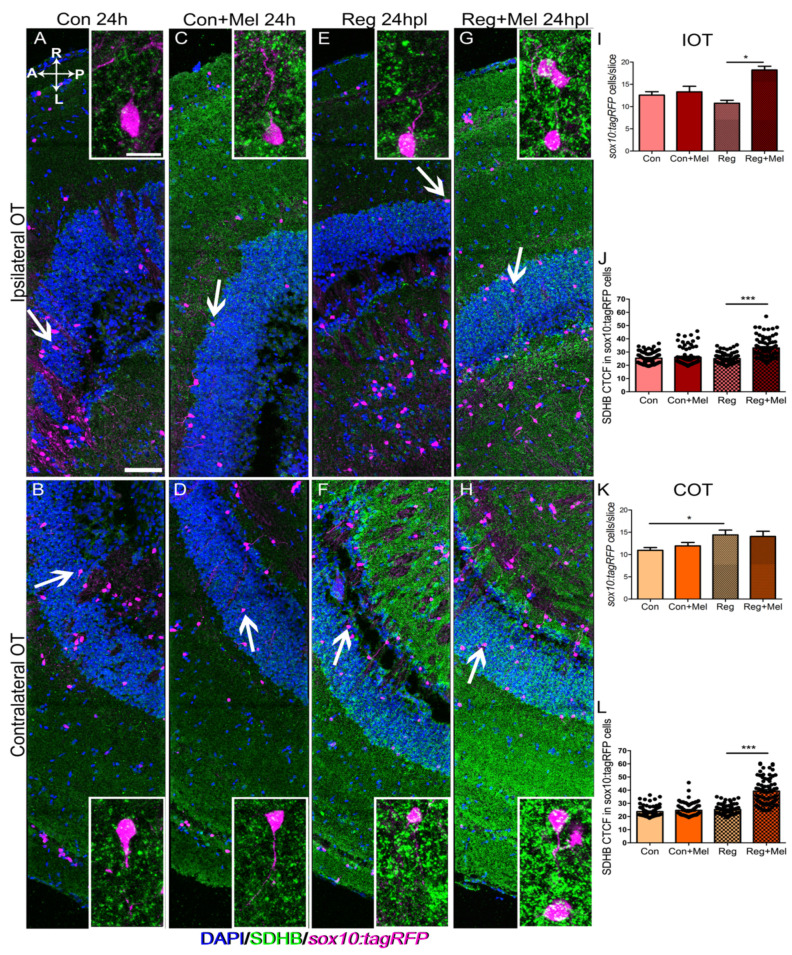
Analysis of oligodendrocyte and the mitochondrial distribution at 24 hpl. Immunostaining for SDHB (green) in a transgenic line carrying *sox10:tagRFP* reporter (magenta). Nuclei are counterstained with DAPI (blue). (**A**–**H**) Control IOT (**A**), control COT (**B**), control exposed to melatonin IOT (**C**), control exposed to melatonin COT (**D**), regenerating fish IOT (**E**), regenerating fish COT (**F**), regenerating fish exposed to melatonin IOT (**G**), regenerating fish exposed to melatonin COT (**H**). (**I**–**L**) Quantification of the number of oligodendrocytes per section in the IOT (**I**) and the COT (**K**); CTCF value of SHDB in oligodendrocytes in the IOT (**J**) and the COT (**L**). A: anterior; Con: control; COT: contralateral optic tectum; h: hours; hpl: hours post-lesion; IOT: ipsilateral optic tectum; L: left; Mel: melatonin; P: posterior; R: right; Reg: regeneration; r.u.: random units. ANOVA with Tukey’s Multiple Comparison Test * *p* < 0.05; *** *p* < 0.001. Scale bar: 100mm; 10mm in inset. n = 4 for each group.

We observed important and unexpected changes in the ROS state in the OT. To further characterize this aspect and investigate if the production of ROS occurred simultaneously with changes in the mitochondria, first, we quantified the number of full oligodendrocytes present in the horizontal section based on the presence of *sox10:tagRFP* and their morphology. We also stained mitochondria using the SDHB antibody and quantified its fluorescence specifically in these oligodendrocytes of the OT (Figure 3. for 3 hpl and Figure 4 for 24 hpl).

Oligodendrocytes were observed all over the horizontal section of the IOT (Figure 3A,C,E,G) and the COT (Figure 3B,D,F,H) from the tectal superficial layers to the PGZ, close to the ventricle. Mitochondria (SDHB staining) are shown in green and, as expected, are present in every cell type (Figure 3). Insets allowed us to explore the mitochondrial content of specific oligodendrocytes (insets in Figure 3).

At 3 hpl, we observed a reduction in the number of *sox10:tagRFP* oligodendrocytes in the IOT and the COT (For IOT: Figure 3A vs. Figure 3E, quantified in Figure 3I; for COT: Figure 3B vs. Figure 3F, quantified in Figure 3K). Melatonin blocked this phenotype in the IOT (Figure 3G, quantified in Figure 3I), but not in the COT (Figure 3H, quantified in Figure 3K). Exposure to melatonin produced an increase in SDHB fluoresce in control animals (for IOT: Figure 3A vs. Figure 3C, quantified in Figure 3J; for COT: Figure 3B vs. Figure 3D, quantified in Figure 3L). The oligodendrocytes of both OT hemispheres in regenerating animals showed a decreased fluorescence for mitochondria, suggesting a reduction in the population (for IOT: Figure 3E, quantified in Figure 3J; for COT: Figure 3F quantified in Figure 3L). Interestingly, exposure to melatonin in the injured fish abolished the decrease in SDHB fluorescence in both hemispheres of the OT (for IOT: Figure 3G, quantified in Figure 3J; for COT: Figure 3H quantified in Figure 3L). We did not quantify other cells of the OT due the lack of clear cellular landmarks, but a similar trend could be observed: reduce fluorescence in injured fish, suppression of this phenotype by melatonin and little or no changes in control animals (Figure 3A–H).

By 24 h (Figure 4) the number of oligodendrocytes in the IOT is almost the same in the control and regenerating fish (Figure 4A vs. Figure 4E, quantified in Figure 4I). Melatonin increased the number of oligodendrocytes just in the IOT of lesioned fish (Figure 4C vs. Figure 4G, quantified in Figure 4I). However, regenerating fish presented more *sox10:tagRFP* oligodendrocytes in the COT (Figure 4B vs. Figure 4F, quantified in Figure 4K), with no further increased induced by melatonin (Figure 4F vs. Figure 4H, quantified in Figure 4K). In terms of SDHB fluorescence, an indirect view of the mitochondrial population, we could observe a similar effect. There was no difference within the control group or between the control and regenerating fish (Figure 4A–F, quantified in Figure 4J for IOT and Figure 4L for COT). However, the oligodendrocytes in the injured fish exposed to melatonin presented higher SDHB fluorescence (Figure 4G,H, quantified in Figure 4J for IOT and Figure 4L for COT). These data indicate changes in the oligodendrocytes concomitant to changes in their mitochondrial population.

### 3.4. Exposure to Melatonin Induces Changes in the OT Undergoing Regeneration

We have described increased ROS production after ON crushing, together with a decrease in the number of *sox10:tagRFP* oligodendrocytes and their mitochondria in the OT at 3 hpl. Both effects were blocked by exposure to melatonin. Thus, we wanted to know if melatonin had a short-term impact on the general regeneration of the visual system. Acetylcholine is one of the most important neurotransmitters in the visual system [38]. The activity of acetylcholine depends on two enzymes, one for its production: choline acetyl transferase (ChAT); and another for its degradation: acetylcholine esterase (AChE). We explored both at 72 hpl through immunohistochemistry and histochemistry, respectively.

**Figure 5:**
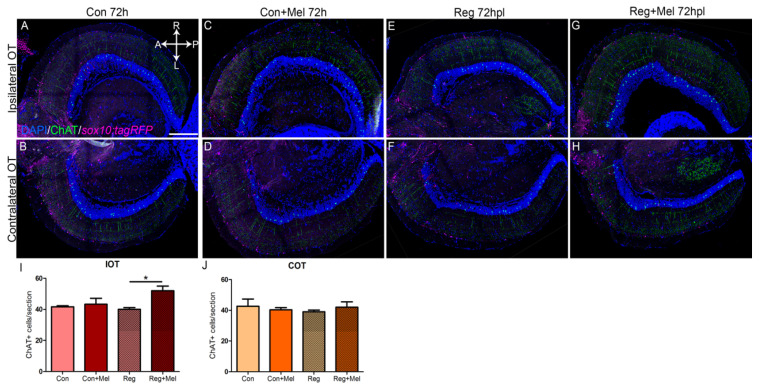
Quantification of ChAT neurons during regeneration at 72 hpl. (**A**–**H**) Immunohistochemistry against ChAT in the OT at 72 hpl in control (**A**,**B**), control exposed to melatonin (**C**,**D**), injured (**E**,**F**) and injured treated with melatonin (**G**,**H**). (**I**,**J**) Quantification of ChAT neurons per section of IOT (**I**) and COT (**J**). A: anterior; ChAT: choline acetyltransferase; Con: control; COT: contralateral optic tectum; h: hours; hpl: hours post-lesion; IOT: ipsilateral optic tectum; L: left; Mel: melatonin; P: posterior; R: right; Reg: regeneration; r.u.: random units. ANOVA with Tukey’s Multiple Comparison Test * *p* < 0.05. Scale bar: 200 mm. n = 4 for each group.

ChAT neurons were present in the PGZ projecting towards more superficial areas of the OT. We found around forty of these neurons per section of OT in both hemispheres in control animals (Figure 5A,B, quantified in Figure 5I,J). As expected, exposure to melatonin did not induce any changes in control animals (Figure 5C,D, quantified in Figure 5I,J). At this stage, fish undergoing regeneration did not show any differences either (Figure 5C,D, quantified in Figure 5I,J). However, we found more ChAT neurons in the IOT of fish that were injured and exposed to melatonin (Figure 5G, quantified in Figure 5I) but not in the COT (Figure 5H, quantified in Figure 5J). The IOT is the only hemisphere receiving light input at this timepoint.

**Figure 6:**
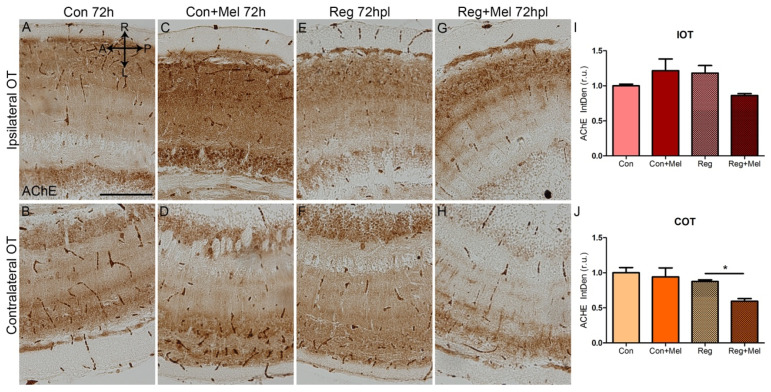
Quantification of AChE during regeneration at 72 hpl. (**A**–**H**) Histochemistry for AChE in the OT at 72 hpl in control (**A**,**B**), control exposed to melatonin (**C**,**D**), injured (**E**,**F**) and injured treated with melatonin (**G**,**H**). (**I**,**J**) Quantification of AChE staining in the IOT (**I**) and COT (**J**). A: anterior; AChE: acetylcholine esterase; Con: control; COT: contralateral optic tectum; h: hours; hpl: hours post-lesion; IOT: ipsilateral optic tectum; L: left; Mel: melatonin; P: posterior; R: right; Reg: regeneration; r.u.: random units. ANOVA with Tukey’s Multiple Comparison Test * *p* < 0.05. Scale bar: 200 mm. Scale bar: 200 mm. n = 4 for each group.

To follow up on the impact of melatonin on the acetylcholine metabolism, we analyzed the activity of the AChE through histochemistry (Figure 6). We did not find significant differences in the staining between control, control exposed to melatonin and injured fish in either side of the OT (Figure 6A–F, quantified in Figure 6I for IOT and in Figure 6J for COT). However, we found a decreased activity of AChE in the COT of lesioned fish treated with melatonin (Figure 6H, quantified in Figure 6J). This is the side that is responding to injury and thus is not receiving light input. We did not find differences in the IOT of regenerating fish treated with melatonin (Figure 6G, quantified in Figure 6I).

### 3.5. Melatonin Treatment Hinders Regeneration

**Figure 7:**
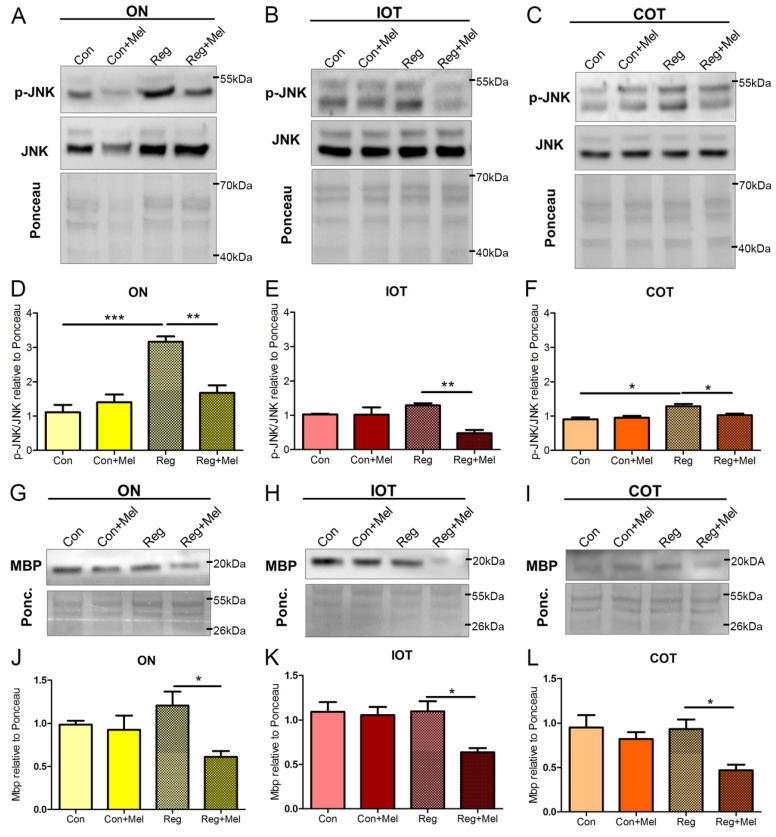
Quantification of MBP at 72 hpl. (**A**–**F**) Western blot for p-JNK and JNK in tissue from ON (**A**), IOT (**B**), COT (**C**). Quantifications represent value of p-JNK relative to total JNK and relative to total protein (Ponceau), quantified in ON (**D**), IOT (**E**), COT (**F**). (**G**–**L**) Western blot for MBP in tissue from ON (**G**), IOT (**H**), COT (**I**). Quantifications represent value of MBP relative to total protein (Ponceau) in ON (**J**), IOT (**K**), COT (**L**). Con: control; COT: contralateral optic tectum; hpl: hours post-lesion; IOT: ipsilateral optic tectum; MBP: Myelin Basic Protein; Mel: melatonin; ON: optic nerve; Reg: regeneration; r.u.: random units. ANOVA with Tukey’s Multiple Comparison Test was performed to search for differences among all groups * *p* < 0.05; ** *p* < 0.01; *** *p* < 0.001. n = 4 for each group.

We wondered if the increased ROS production in injured fish (Figure 1B,C) and the exposure to melatonin (Figure 2A–C) had an impact on cell signaling. Thus, we investigated the abundance of JNK, a MAP Kinase involved in stress response and its active form p-JNK [39]. At 3 hpl, regenerating fish had an increased p-JNK/JNK ratio in ON (Figure 7A, quantified in Figure 7D) and the COT (Figure 7C, quantified in Figure 7F), the areas affected by the injury. Melatonin treatment blocked this upregulation in both regions (ON: Figure 7A, quantified in Figure 7D; COT: Figure 7C, quantified in Figure 7F). In the IOT we did not observe a significant increase in p-JNK/JNK (Figure 7B, quantified in Figure 7E); but this ratio was also reduced in this area after melatonin treatment (Figure 7B, quantified in Figure 7E). IOT was the only visual area where melatonin had a clear antioxidant effect at 3 hpl (Figure 2B). Melatonin did not alter the p-JNK/JNK ratio in control animals (Figure 7A–C, quantified in Figure 7D–F). Thus, we observed an activation of the JNK pathway after injury that was quenched by melatonin in all three areas.

Exposure to melatonin impacts signaling, mitochondrial state and different cell populations. Therefore, we wondered if the general recovery of the injured fish was affected. We then analyzed the presence of MBP in all three visual areas by Western blot at 72 hpl (Figure 7G–L). Melatonin did not produce any impact on the abundance of MBP in control animals (Figure 7G–I, quantified in Figure 7J–L). At this stage, we did not observe any important changes in the abundance of MBP in the regenerating fish (Figure 7G–I, quantified in Figure 7J–L). However, in all areas, injured fish exposed to melatonin presented significantly less MBP (Figure 7G–I, quantified in Figure 7J–L). These results highlight the detrimental impact of melatonin in injured fish.

## 4. Discussion

Our results indicate that ROS are produced three hours after injury in different parts of the visual system, with an important contribution from mature oligodendrocytes. Modulating oxidative stress with melatonin induces an aberrant regeneration including diminished production of myelin and disrupted differentiation of the OT. Furthermore, we show that, not only oligodendrocytes in the ON respond to injury, but also those located in the OT. As described during tail regeneration [20], ROS seem to positively contribute to CNS regeneration in zebrafish.

The role of ROS has been explored during the regeneration of open wounds. After the amputation of part of the zebrafish tail, free radicals are produced for at least 24 h and trigger JNK signaling and apoptosis. Blocking either process has detrimental effects and delays regeneration [20,40]. Similar mechanisms have been also reported during wound healing in *Xenopus* [41], *Drosophila* [42] and *C. elegans* [43], although the type of injury was different. Cellular changes induced by ROS, such as recruitment of adherens junctions, are also conserved in different models [42]. Our results show that ROS production by oligodendrocytes peaks in the visual system early, at 3 hpl, including the OT. A previous work also reported the relevance of ROS in the brain using a model of a stab wound in the telencephalon, although they describe the importance of oxidative stress at 24 hpl [22]. In addition to these small timing differences, our results support the role of free radicals as a fast regenerative signal also in the visual system. The source of these ROS has been searched for by several groups. In open wound models, cells close to the wound produce free radicals [42]. Other cells can also produce ROS, for example microglia, another type of supporting glial cell in the nervous system, respond to the oxidative stress produced by a stab at 6 hpl, and then produce ROS themselves [44]. We have shown that fully differentiated oligodendrocytes (*sox10:EGFP*) become a source of ROS after injury at 3 hpl. ROS can control oligodendrocyte differentiation and activity [23,45]. Thus, it is interesting to hypothesize that oligodendrocytes might be the first responders to the injury in the visual system and become a ROS hub that could attract other cells.

To delve into the role of the oligodendrocytes, we tried to cease the production of free radicals with melatonin. However, a clear antioxidant effect was only observed in non-GFP cells in controls, which proved the antioxidant of the melatonin treatment [24]. Fully differentiated oligodendrocytes (*sox10:EGFP*) seem to behave differently to melatonin, as mitochondrial ROS rise. Concomitantly, melatonin produces an increase in mitochondria per oligodendrocyte. This free radical increment in basal conditions may be due to mitochondrial fission [46]. Melatonin had an even more complex effect on the oligodendrocytes under regenerative conditions since it had an antioxidant effect depending on the timepoint, first in the IOT (3 hpl) and later in the COT (24 hpl). This time-dependent melatonin effect has been previously described in other scenarios and reveals a complex interaction with the oligodendrocyte’s biology [47]. It is not surprising that oligodendrocytes respond to melatonin since they present both types of receptors [48]. In our scenario, the effect of melatonin seems to depend on the cellular environment, which is very different between both hemispheres, one responding directly to the ON injury (COT) and the other probably responding to excessive workload (IOT) since all eye projections are contralateral in zebrafish [37]. It seems clear, however, that melatonin disrupts the normal process during regeneration, for example delaying the loss of mitochondria in both hemispheres of the OT, a phenotype that has been reported previously [49].

We were interested in evaluating the impact of melatonin on the general regenerative process. Thus, we studied the functionality of the OT (acetylcholine pathway) and the myelination. This neurotransmitter system plays important roles in visual processing and perception in zebrafish [38] and mammals [50]. At the timepoints examined by us, there were no changes in this system during regeneration, but melatonin produced profound effects that were hemisphere specific. ChAT neurons were increased in the IOT, the side that is still receiving light, and AChE activity was decreased in the COT, the side that lost the connection to the eye. When ROS balance is altered, Morin, another antioxidant, has been also shown to decrease AChE activity [51]. More interestingly, melatonin has been described to support survival of ChAT neurons [52,53] and to reduce AChE activity in different rodent models [54,55]. The increased number of ChAT neurons could also be related to the reduction of JNK signaling induced earlier, as shown in vitro [56,57]. So, during regeneration there is a specific cellular environment, likely related to the REDOX misbalance and the presence/absence of light input, where melatonin can push the differentiation of ChAT neurons and block AChE activity.

We have reported the activation of JNK pathway in the ON but also far from the wound, in both OT of injured fish. We have also described the negative impact of melatonin in JNK activation during regeneration. It has been shown that ROS spike leads to JNK and P38 Map Kinase activation [42,58]; and the JNK pathway through c-Jun activation is necessary for normal regeneration [40]. Upregulation of the JNK pathway in our model may be linked to the drop of fully differentiated oligodendrocytes observed in regeneration [59]. Melatonin also hindered the recovery of MBP, a protein involved in axon myelination [29]. As far as we know, there is no report linking melatonin and the myelination process itself, but melatonin promotes oligodendrocytic survival and maturation in different pathological scenarios [48,60]. We have also observed an increased number of oligodendrocytes that survived the injury after exposure to melatonin. Thus, the disruption of JNK by melatonin may lead to the accumulation of aberrant oligodendrocytes, as we observed in IOT, and/or a global abnormal regeneration (OT and ON) [40,54]. Death of mature oligodendrocytes is necessary for proper regeneration since surviving oligodendrocytes mistarget new axons and produce aberrant myelination [14].

Several groups have reported the benefits of melatonin during regeneration, at least in the rodent peripheral system [49,61] or during demyelinating diseases [62,63]. In fact, melatonin may improve the function of Schwann cell (another type of myelinating cell) through a mechanism related to mitochondrial biology and its antioxidant properties [61,64]. However, other authors have reported that ROS are beneficial for the repairment of the peripheral nervous system [65] and during the regeneration of mammalian fat pads [66] and liver [67], and blocking ROS during zebrafish tail regeneration is detrimental [20,21]. All of them tissues with important regenerative capabilities. Then, there could exist a relationship between tissues’ capacity to respond to ROS and their regenerative potential. Our results support the beneficial effects of ROS during the regeneration of the zebrafish visual system and the detrimental impact of melatonin. Caution might be advised with the therapeutic potential of this molecule. In some scenarios, melatonin might be beneficial but its impact on neural differentiation is not well understood in pathological conditions. For example, it can exacerbate neurological disability scores in rodents with encephalomyelitis [68]. More experiments are necessary to understand the role of ROS during the regeneration of different tissues and models, and to comprehend the precise mechanisms elicited by melatonin.

## 5. Conclusions

Oligodendrocytes present important levels of ROS production. ROS are produced during early regeneration in the oligodendrocytes of the ON and the OT. Deregulation of the ROS production leads to aberrant regeneration. Exposure to melatonin is detrimental to the regeneration of the zebrafish visual system.

## Figures and Tables

**Table 1:** Primary antibodies.

Antigen	Host	Reference	Dilution	Observations
Choline acetyltransferase (ChAT)	Goat	Sigma-Aldrich; ab144P	1:100	Catalyzes the reversible synthesis of acetylcholine from acetyl CoA and choline at cholinergic synapses
Succinate dehydrogenase complex subunit B (SDHB)	Mouse	Abcam; ab14714	1:200	Complex II of the respiratory chain involved in the oxidation of succinate

**Table 2:** Secondary antibodies.

Antigen	Host	Reference	Conjugated	Dilution
Anti-Goat	Donkey	Jackson ImmunoResearch (West Grove, PA, USA)	Alexa 488	1:400
Anti-Mouse	Donkey	Jackson ImmunoResearch	Alexa 647	1:400

## Data Availability

Data are contained within the article.

## Supplementary Materials

The following supporting information can be downloaded at: https://www.mdpi.com/article/10.3390/antiox12122026/s1, Figure S1: Analysis of the mitochondrial membrane potential.title (A) Membrane potential measured with MitoProbe DiIC1^®^ by flow cytometry. Con: control; COT: contralateral optic tectum; hpl: hours post-lesion; IOT: ipsilateral optic tectum; ON: optic nerve; Reg: regeneration; r.u.: random units.
