# Supplementary material for: REDOX Balance in Oligodendrocytes Is Important for Zebrafish Visual System Regeneration
Source: Antioxidants (Basel). 2023 Nov 22;12(12):2026. doi: 10.3390/antiox12122026 (PMC10740785; doi:10.3390/antiox12122026)
Supplement: Supplementary file 1 [file antioxidants-12-02026-s001.zip › antioxidants-2695512-supplementary.pdf]

antioxidants

MDPI

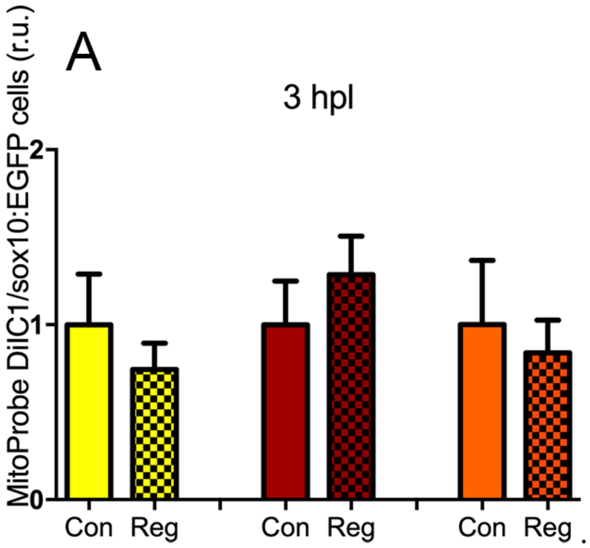

Figure S1. Analysis of the mitochondrial membrane potential. A) Membrane potential measured with MitoProbe DiIC1® by flow cytometry. Con: control; COT: contralateral optic tectum; hpl: hours post-lesion; IOT: ipsilateral optic tectum; ON: optic nerve; Reg: regeneration; r.u.: random units.
